# Roxadustat Activates Thyroid Hormone Receptors α and β at Clinically Relevant Concentrations

**DOI:** 10.7759/cureus.89750

**Published:** 2025-08-10

**Authors:** Koji Morita, Toru Iigo, Kaho Jimbo, Akihiro Jimbo, Takashi Suzuki, Naoki Edo, Hiroki Yamazaki, Kenji Uno, Kazuhisa Tsukamoto, Toshio Ishikawa

**Affiliations:** 1 Division of Endocrinology and Metabolism, Department of Internal Medicine, Teikyo University School of Medicine, Tokyo, JPN

**Keywords:** hypoxia-inducible factor-prolyl hydroxylase inhibitor, luciferase assay, roxadustat, thyroid hormone receptors, thyromimetic activity

## Abstract

Roxadustat, a drug recently approved for treating renal anemia, might activate thyroid hormone receptors (TRα and TRβ). We, thus, investigated the direct effects of roxadustat on intact TRα and TRβ in vitro.

U2OS, MCF-7, COS-7, and Caco-2 cells were transiently transfected with a firefly luciferase reporter plasmid highly responsive to the activation of TRα and TRβ, along with a plasmid overexpressing either TRα or TRβ. Transfected cells were incubated for three hours in 100% thyroid hormone-stripped fetal bovine serum (FBS) with or without either roxadustat or triiodothyronine (T3). Also, transfected U2OS cells were placed for three hours in 100% unprocessed normal adult human serum with or without the addition of either roxadustat or T3. Cell lysates were measured for firefly luciferase activity.

Roxadustat induced firefly luciferase activities when added at clinically achievable serum concentrations in TRα- or TRβ-transfected U2OS, MCF-7, COS-7, and Caco-2 cells placed in thyroid hormone-deprived FBS. The induction was comparable to or greater than that observed with T3 added at normal or even supranormal serum concentrations. Roxadustat also increased firefly luciferase activities in TRα- or TRβ-transfected U2OS cells in adult human serum.

These data suggest the possibility that roxadustat could activate TRα and TRβ at clinically relevant concentrations. Physicians prescribing roxadustat should be aware of its thyromimetic activity.

## Introduction

Thyroid hormones (thyroxine (T4) and its active derivative triiodothyronine (T3)) exert pleiotropic actions on numerous tissues via thyroid hormone receptors (TRs), namely, TRα and TRβ encoded by *THRA* and *THRB*, respectively. Depending on tissues, either TRα or TRβ is predominantly expressed; for example, the heart and bone mainly express TRα, whereas the pituitary and liver predominantly express TRβ [1]. T3 increases heart rate and bone resorption primarily through TRα, while it suppresses thyroid-stimulating hormone (TSH) secretion and affects hepatic lipid metabolism via TRβ [1]. Therefore, chronic TRα overactivation may cause tachyarrhythmia and osteoporosis, while persistent TRβ stimulation may reduce serum low-density lipoprotein cholesterol (LDL-C) and hepatic fat content. These clinically beneficial TRβ-mediated effects prompted researchers to develop TRβ-specific agonists [2-4]. In fact, the US Food and Drug Administration recently approved resmetirom, a TRβ-selective agonist, for the treatment of noncirrhotic nonalcoholic steatohepatitis (NASH) with moderate to advanced liver fibrosis [4].

Interestingly, a drug being prescribed for a disorder unrelated to thyroid dysfunction may stimulate TRs. Roxadustat, an orally given hypoxia-inducible factor-prolyl hydroxylase (HIF-PH) inhibitor [5], has become available for the treatment of renal anemia in the EU, China, Japan, etc. This drug is structurally similar to T3 and may activate TRs [6]. However, it has not been thoroughly investigated whether roxadustat activates TRs at a concentration that can occur in the serum of patients on this drug. Thus, in this study, we tested whether roxadustat could activate TRs at clinically relevant concentrations by performing reporter gene assays.

This article was previously posted to the Research Square preprint server (Preprint: Morita K, Uchino T, Edo N, Uno K, Tsukamoto K, Ishikawa T. Roxadustat Activates Thyroid Hormone Receptors α and β at Clinically Relevant Concentrations. April 27, 2022).

## Materials and methods

Cell culture

Cell lines with high transfection efficiency were used. Human osteosarcoma U2OS cells were purchased from KAC (Kyoto, Japan), human mammary carcinoma MCF-7 cells and African green monkey kidney-derived COS-7 cells from the JCRB cell bank (Osaka, Japan), and human colorectal carcinoma Caco-2 cells from DS Pharma Biomedical (Suita, Japan). All cell lines were maintained in Dulbecco’s modified Eagle’s medium (DMEM) containing antibiotics and 9% (v/v) heat-inactivated fetal bovine serum (FBS). During the experiments, cells were placed either in 100% heat-inactivated FBS that had been deprived of thyroid hormone by pretreatment with Amberlite^®^ IRA-400 Cl (Merck, Darmstadt, Germany) [7,8] or in 100% unprocessed adult human serum. These environments were chosen because they were closer to the in vivo setting than the FBS-containing DMEM.

Chemicals

Roxadustat and molidustat (another HIF-PH inhibitor) were obtained from Cayman Chemical (Ann Arbor, MI, USA), and T3 was purchased from Merck. Dimethyl sulfoxide (DMSO) was used as a solvent control (final concentration: 0.1% (v/v)). Roxadustat and molidustat were added to cells at 1, 3, 6, 15, or 30 μg/mL and at 1 μg/mL, respectively, based on their serum concentrations observed in patients (up to 18 and 1.2 μg/mL for roxadustat and molidustat, respectively, according to their prescribing information (Astellas Pharma Inc., Tokyo, Japan; Bayer Yakuhin Ltd., Osaka, Japan)). Considering the concentration of total T3 in normal human serum (0.8-1.6 ng/mL), 1, 2, 5, or 10 ng/mL T3 was added to thyroid hormone-stripped FBS or adult human serum.

Plasmids

Human TRα1 and TRβ1 expression vectors (named pCI-hTRα and pCI-hTRβ, respectively) were constructed as previously reported [9]. The pCI-neo Mammalian Expression Vector (hereafter abbreviated as pCI; Promega, Madison, WI, USA) was used as the empty control vector. Characterization of the T3-responsive firefly luciferase reporter gene (which was named 12DR4-luc2CP) and the *Renilla* luciferase reporter gene phRL-CMV used as an internal control was also described previously [9]. The sequence of 12DR4-luc2CP is shown in the appendix.

Reporter gene assays

One 10-cm dish of cells (about 80% confluent) was transfected with 5.0 μg of pCI-hTRα, pCI-hTRβ or pCI, 5.0 μg of 12DR4-luc2CP, and 50 ng of phRL-CMV using FuGENE HD (Promega). Transfected cells were seeded equally into one 96-well Black & White tissue culture plate (Perkin-Elmer, Waltham, MA, USA) in either 100% thyroid hormone-deprived FBS or 100% adult human serum. Depending on the well number needed in each experiment, the amounts of cells and plasmids were up- or down-scaled proportionally. The next day, the transfected cells were treated as indicated (three or four wells were assigned to each treatment) for three hours, lysed, and measured for firefly and *Renilla* luciferase activities using the Dual-Luciferase Assay System (Promega) in the AB-2350 Phelios microplate luminometer (ATTO, Tokyo, Japan). Normalized firefly luciferase activity (ratio of firefly/*Renilla* luciferase luminescence) was used for analysis.

Statistical analysis

One-way analysis of variance (ANOVA) followed by Tukey’s post hoc test was performed, using GraphPad Prism version 6.0 for Windows (GraphPad Software, San Diego, CA, USA). A p-value < 0.05 was considered significantly different.

## Results

**Figure 1:**
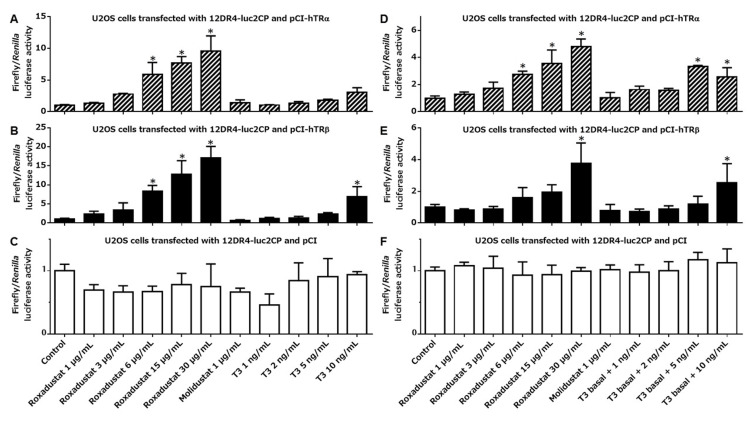
Roxadustat-induced TR activation in U2OS cells U2OS cells transfected with 12DR4-luc2CP along with pCI-hTRα (A and D), pCI-hTRβ (B and E), or pCI (C and F) were treated for 3 h in either 100% thyroid hormone-stripped FBS (A-C) or 100% unprocessed human serum (D-F), as indicated at the bottom of graphs (C and F). Columns and error bars represent means and standard deviations, respectively, of triplicate or quadruplicate samples. The mean value for the control cells was defined as 1.0. An asterisk indicates a significantly higher value than that of the control. TR: thyroid hormone receptor; FBS: fetal bovine serum

**Figure 2:**
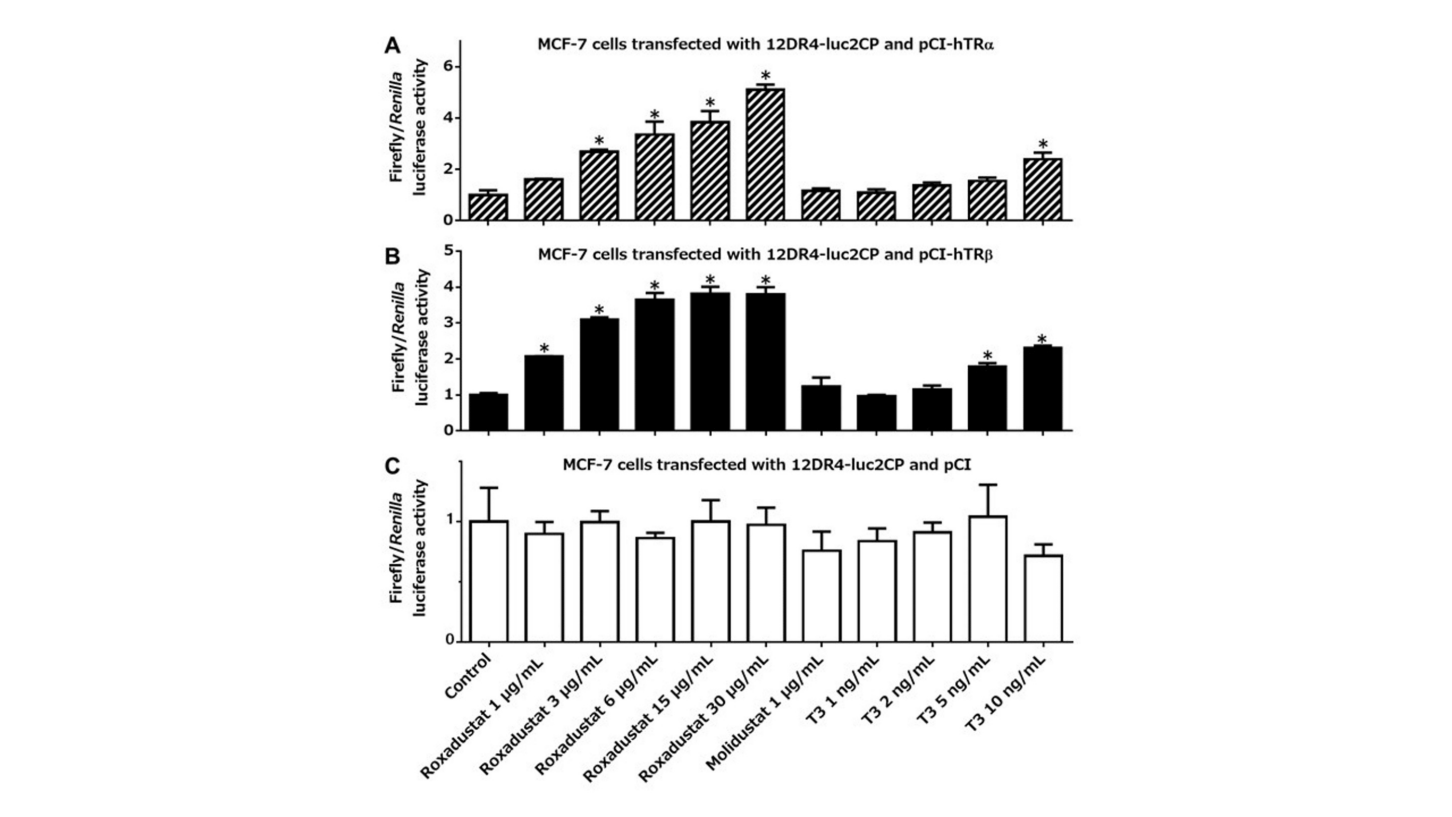
Roxadustat-induced TR activation in MCF-7 cells MCF-7 cells transfected with 12DR4-luc2CP along with pCI-hTRα (A), pCI-hTRβ (B), or pCI (C) were treated for 3 h in 100% thyroid hormone-stripped FBS, as indicated at the bottom of graph (C). Columns and error bars represent means and standard deviations, respectively, of triplicate or quadruplicate samples. The mean value for the control cells was defined as 1.0. An asterisk indicates a significantly higher value than that of the control. TR: thyroid hormone receptor; FBS: fetal bovine serum

**Figure 3:**
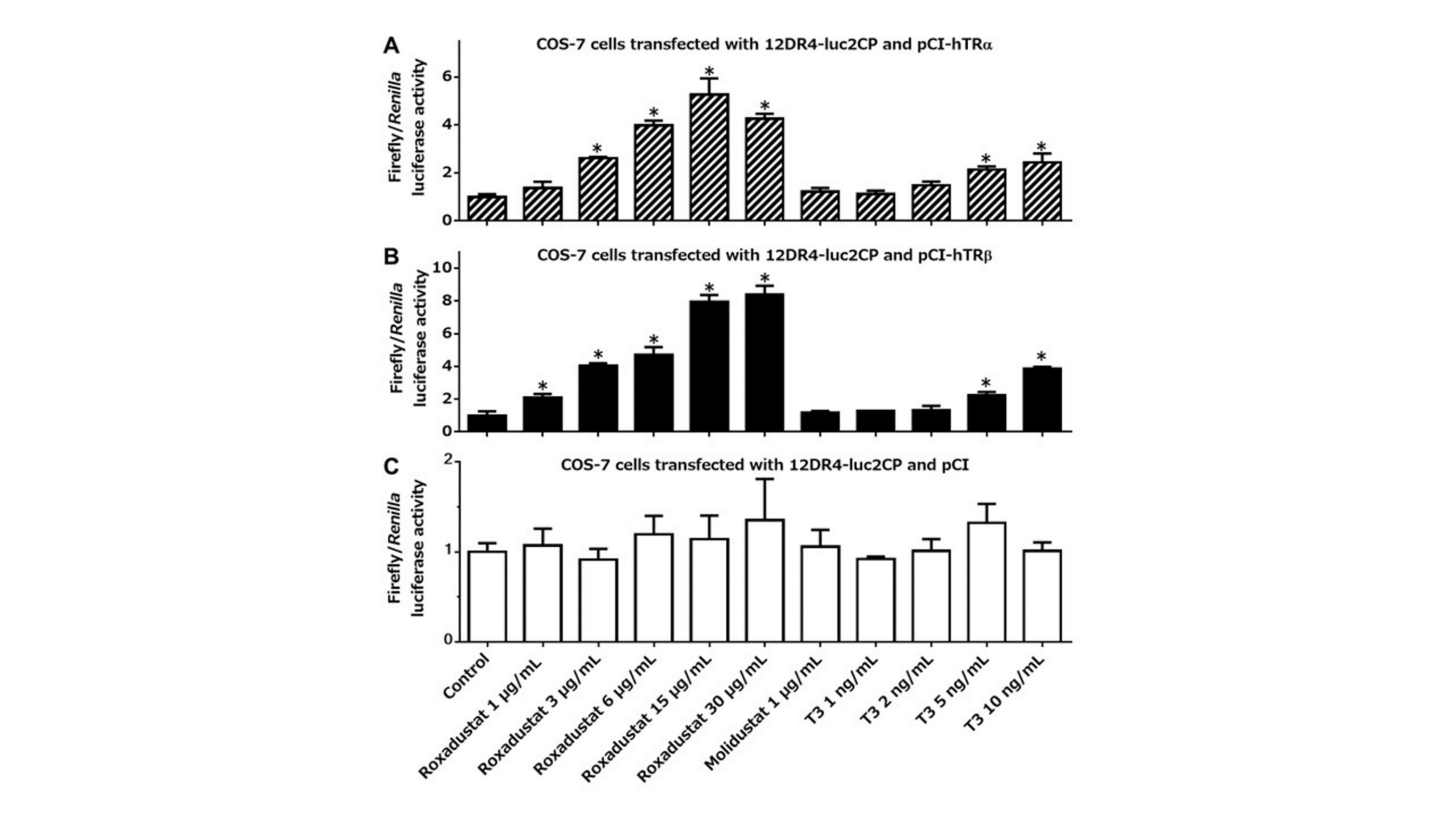
Roxadustat-induced TR activation in COS-7 cells COS-7 cells transfected with 12DR4-luc2CP along with pCI-hTRα (A), pCI-hTRβ (B), or pCI (C) were treated for 3 h in 100% thyroid hormone-stripped FBS, as indicated at the bottom of graph (C). Columns and error bars represent means and standard deviations, respectively, of triplicate or quadruplicate samples. The mean value for the control cells was defined as 1.0. An asterisk indicates a significantly higher value than that of the control. TR: thyroid hormone receptor; FBS: fetal bovine serum

**Figure 4:**
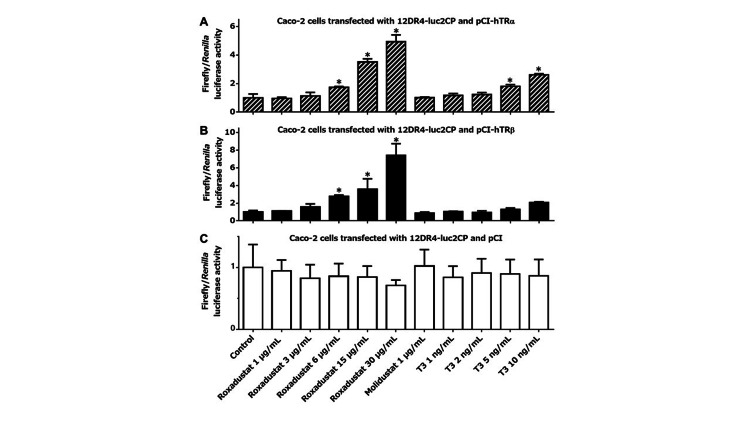
Roxadustat-induced TR activation in Caco-2 cells Caco-2 cells transfected with 12DR4-luc2CP along with pCI-hTRα (A), pCI-hTRβ (B), or pCI (C) were treated for 3 h in 100% thyroid hormone-stripped FBS, as indicated at the bottom of graph (C). Columns and error bars represent means and standard deviations, respectively, of triplicate or quadruplicate samples. The mean value for the control cells was defined as 1.0. An asterisk indicates a significantly higher value than that of the control. TR: thyroid hormone receptor; FBS: fetal bovine serum

We transfected 12DR4-luc2CP, along with pCI-hTRα, pCI-hTRβ, or pCI, into U2OS cells and then treated them as indicated for three hours in 100% thyroid hormone-stripped FBS. In cells transfected with either pCI-hTRα (Figure 1A) or pCI-hTRβ (Figure 1B), roxadustat increased firefly luciferase transcription dose-dependently, whereas another HIF-PH inhibitor molidustat, which is structurally dissimilar to T3, did not. Of note, roxadustat in the range of therapeutic serum concentrations (6 or 15 μg/mL) stimulated TRα- or TRβ-mediated gene transcription to an extent comparable to or exceeding the effect of supranormal serum levels of T3 (5 or 10 ng/mL). By contrast, in cells expressing only endogenous TRs, no significant induction was observed with any treatment (Figure 1C). We then treated transfected U2OS cells with roxadustat in 100% raw adult human serum, which might provide a condition even closer to the extracellular environment in the body of roxadustat-treated patients (Figures 1D-1F). As compared to when thyroid hormone-deprived FBS was used, the presence of T3 in unprocessed serum blunted the fold increase in TR activation after roxadustat treatment. Nonetheless, 15 μg/mL roxadustat still tended to enhance TR-driven transcription, at least to a comparable degree to T3, which was 5 ng/mL above the normal serum T3 level. We also tested the thyromimetic activity of roxadustat in other cell lines. Similar tendencies, although not so striking as in U2OS cells, were clearly observed in MCF-7, COS-7, and Caco-2 cells cultured in thyroid hormone-stripped FBS (Figures 2-4). Taken together, our experiments suggest that roxadustat is a TRα and TRβ activator that is comparable to or possibly even stronger than T3.

## Discussion

Clinically, it has been documented that roxadustat may affect the thyroid hormone axis. For example, in Japan, the prescribing information for roxadustat clearly states that this drug may cause hypothyroidism (which apparently means low serum free T4 and free T3) and reduce serum TSH and LDL-C. Also, several case reports [10-15], cohort studies, systematic reviews, and meta-analyses [16-24] regarding the effects of roxadustat on thyroid function have been published in the PubMed database. One cohort study demonstrated that patients initiated on roxadustat were more likely to experience a reduction in TSH levels to less than half of baseline compared to those receiving erythropoietin [17]. Other studies reported a correlation between the extent of serum LDL-C and TSH reductions following roxadustat treatment [15,21], which may be attributable to TRβ activation induced by roxadustat. This thyroid dysfunction, uniquely associated with roxadustat and not observed with other HIF-PH inhibitors, appears to be reversible after discontinuation of roxadustat [10-12,14,15].

As for basic research, Yao et al. published a report on roxadustat’s thyromimetic action [6]. They transfected a fusion protein (which consisted of the TRα or TRβ ligand-binding domain (LBD) and the GAL4 DNA-binding domain) into cells and stimulated them with roxadustat for 24 hours in DMEM containing 10% (v/v) FBS. They showed that roxadustat, at concentrations as low as 1 nM (0.35 ng/mL), induced a detectable increase in transcriptional activity of the LBD of TRα or TRβ. Our study strengthens their findings in the following points. First, we demonstrated the activation of the intact TRα and TRβ, instead of their LBDs, by roxadustat. Second, we observed reporter gene induction just after three hours of treatment, indicating that TR activation was caused by roxadustat per se, but not by its cellular metabolites, nor as a result of some roxadustat-triggered pathways that would become apparent much later (e.g., HIF-PH inhibition). Finally, we showed that roxadustat, at concentrations observed in patients, activated TRs in cells cultured in 100% serum, an environment closer to the in vivo extracellular environment than the regular culture medium. Therefore, our findings strongly suggest that roxadustat’s thyromimetic activity can have a significant clinical impact.

Yao et al. also suggested that roxadustat might act predominantly as a TRβ-selective agonist, because the structural difference between T3 and roxadustat molecules would make it difficult for roxadustat to fit into the binding pocket of TRα [6]. However, aside from roxadustat’s TSH-suppressing and lipid-lowering properties that can be explained by TRβ activation in the pituitary gland and liver, a slight but significant increase in pulse rate and blood pressure was observed in a patient following roxadustat treatment [10], which indicates that roxadustat may exert positive chronotropic and inotropic effects via TRα activation in the heart. Intriguingly, to our knowledge, bradycardia has not been reported in patients with roxadustat-induced hypothyroidism, possibly because of the TRα-mediated chronotropic effect of roxadustat. This lack of bradycardia despite hypothyroidism aligns well with our in vitro observation that roxadustat stimulates not only TRβ but also TRα.

Limitations

We have conducted research using only cultured cells, without evaluating the thyromimetic effects of roxadustat in animal models or prospective, controlled clinical trials. Future research is needed to elucidate how thyroid hormone-responsive factors, such as sex hormone-binding globulin (SHBG), are modulated by the administration of roxadustat in both animals and humans. However, even within the scope of in vitro cellular experiments, there remains room for further investigation. For example, although we have demonstrated roxadustat’s thyromimetic effects in some diverse cell types and consider it highly likely that similar effects occur in others, this does not guarantee that the phenomenon will be observed universally across all cell types. Furthermore, it remains unclear whether roxadustat influences noncanonical TR signaling pathways induced by T3 [25] or affects T3-activated pathways that are independent of TRs [26]. Issues such as these warrant further investigation.

## Conclusions

Using a highly sensitive reporter gene, we have demonstrated that roxadustat induced TR-dependent gene transcription when added at clinically relevant concentrations in TRα- or TRβ-overexpressing U2OS, MCF-7, COS-7, and Caco-2 cells placed in thyroid hormone-deprived FBS. The induction was equal to or greater than that observed with T3 added at normal or even supranormal serum concentrations. Roxadustat also increased TR-mediated reporter gene expression in TRα- or TRβ-overexpressing U2OS cells cultured in adult human serum, which more closely replicates an “in vivo-like” environment than FBS. These data suggest that roxadustat does activate both TRα and TRβ, consistent with clinical observations reported thus far. Physicians should be aware that mild thyrotoxic symptoms as well as low serum TSH levels might be observed in patients being treated with roxadustat, due to its thyromimetic activity.

## Figures and Tables

**Figure 5:**
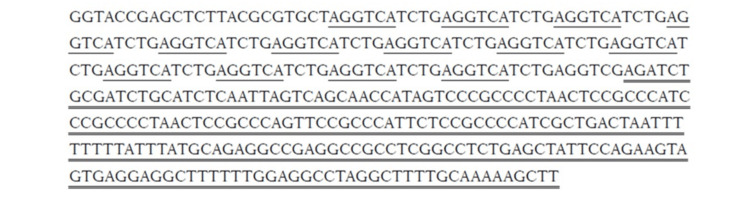
Sequence of 12DR4-luc2CP The 12DR4-luc2CP construct was generated by ligating the Kpn I-Hind III DNA fragment shown here into the pGL4.12 [luc2CP] Vector (Promega). This sequence contains 12 DR4 motifs (AGGTCANNNNAGGTCA), which serve as consensus binding sites for both TRα and TRβ. The underlined sequences represent the AGGTCA half-sites of the DR4 motifs. The double-underlined region is derived from the pGL3-Promoter Vector (Promega).
